# Alpha-adrenergic receptor activation after fetal hypoxia-ischaemia suppresses transient epileptiform activity and limits loss of oligodendrocytes and hippocampal neurons

**DOI:** 10.1177/0271678X231153723

**Published:** 2023-01-26

**Authors:** Simerdeep K Dhillon, Eleanor R Gunn, Mette V Pedersen, Christopher A Lear, Guido Wassink, Joanne O Davidson, Alistair J Gunn, Laura Bennet

**Affiliations:** 1 Department of Physiology, The University of Auckland, Auckland, New Zealand; 2 Department of Pediatrics, Aarhus University Hospital, Aarhus, Denmark

**Keywords:** Hypoxia-ischaemia, sympathetic nervous system, cerebral hypoperfusion, neuro-inhibition, neural injury

## Abstract

Exposure to hypoxic-ischaemia (HI) is consistently followed by a delayed fall in cerebral perfusion. In preterm fetal sheep this is associated with impaired cerebral oxygenation, consistent with mismatch between perfusion and metabolism. In the present study we tested the hypothesis that alpha-adrenergic inhibition after HI would improve cerebral perfusion, and so attenuate mismatch and reduce neural injury. Chronically instrumented preterm (0.7 gestation) fetal sheep received sham-HI (n = 10) or HI induced by complete umbilical cord occlusion for 25 minutes. From 15 minutes to 8 hours after HI, fetuses received either an intravenous infusion of a non-selective alpha-adrenergic antagonist, phentolamine (10 mg bolus, 10 mg/h infusion, n = 10), or saline (n = 10). Fetal brains were processed for histology 72 hours post-HI. Phentolamine infusion was associated with increased epileptiform transient activity and a greater fall in cerebral oxygenation in the early post-HI recovery phase. Histologically, phentolamine was associated with greater loss of oligodendrocytes and hippocampal neurons. In summary, contrary to our hypothesis, alpha-adrenergic inhibition increased epileptiform transient activity with an exaggerated fall in cerebral oxygenation, and increased neural injury, suggesting that alpha-adrenergic receptor activation after HI is an important endogenous neuroprotective mechanism.

## Introduction

Preterm birth is highly associated with mortality and life-long neurodevelopmental disabilities,^1^ and there are no established neuroprotection or neuro-repair interventions. Antenatal and/or perinatal hypoxia-ischaemia (HI) remains a major contributor in the multifactorial aetiology of preterm brain damage.^2,3^ Thus, better understanding of the pathogenesis of HI injury in the preterm brain is vital to improve care of this vulnerable group. It is well established that HI brain injury evolves over hours to days, with characteristic neurophysiological, cerebral perfusion and oxygenation changes over time.^4^ After acute HI there can be transient normalisation of cerebral oxidative metabolism, and perfusion followed by a delayed fall during the early recovery (‘latent’) phase after HI, before the delayed failure of oxidative metabolism and seizures. This delayed or secondary cerebral hypoperfusion after HI has been reported in multiple species and paradigms; the speed of onset and duration of hypoperfusion are broadly associated with the severity of the initial insult.^4^ Secondary hypoperfusion is associated with overall suppression of EEG activity and cerebral metabolism.^5,6^ However, there can be mismatch between cerebral perfusion and metabolism during this phase.

In preterm fetal sheep, cerebral oxygenation measured by near-infrared spectroscopy (NIRS) fell during the phase of delayed hypoperfusion after HI induced by 25 minutes of complete umbilical cord occlusion.^7,8^ Interestingly, in this paradigm, abnormal epileptiform transient activity (spikes, sharp and slow waves) on a suppressed background was present throughout the latent phase, and the frequency of sharp waves was associated with subcortical neuronal loss.^7,9^ The period of cerebral hypoxia corresponded with the peak frequency of abnormal epileptiform transients, raising the possibility that it may represent a second “hit” after HI.^7^ Both in term neonates with HIE and more generally in preterm infants, altered neuro-vascular coupling and low cerebral oxygen saturation in the early period after birth are associated with adverse outcomes.^10,11^

Cerebral hypoperfusion after HI may be mediated by endothelial dysfunction secondary to increased production of reactive oxygen species, altered balance between vasoconstrictors and vasodilators, and inflammatory mediators.^12,13^ However, studies attempting to reverse hypoperfusion using vasodilators have had variable outcomes.^13,14^ For example, in preterm fetal sheep intravenous (i.v.) infusion of an endothelin receptor antagonist (Bosentan) and a vasodilator (L-arginine, a nitric oxide donor) from 30 minutes to 8 hours after the end of HI did not reverse the post-hypoxic hypoperfusion in the superior mesenteric artery bed.^15^ In contrast, i.v. infusion of the non-selective alpha-adrenergic receptor phentolamine reversed the delayed post-hypoxic hypoperfusion in peripheral beds.^15,16^ These data suggest that sympathetic nervous system (SNS) activation is a key mediator of delayed hypoperfusion after HI.

In this study, we examined if alpha-adrenergic receptor blockade in 0.7 gestation preterm fetal sheep after HI induced by complete umbilical cord occlusion for 25 minutes would restore cerebral perfusion, and so improve cerebral oxygenation and reduce neural injury.

## Methods

### Ethical approval

All procedures were approved by the Animal Ethics Committee of the University of Auckland following the New Zealand Animal Welfare Act 1999, and carried out in accordance with the code of Animal ethical conduct established by the Ministry of Primary industries of New Zealand for the use of animals for teaching and research (AEC approval number 1942). The experiments are reported in accordance with the ARRIVE guidelines for reporting animal research.^17^

### Fetal surgery

Thirty Romney-Suffolk cross fetal sheep were instrumented on gestation days 99–100, as previously described.^18^ For twin pregnancies, only one fetus was surgically instrumented. Ewes were acclimatised to laboratory conditions for one week before the surgery, during which time, regular veterinary and welfare checks were performed. Food, but not water, was withdrawn 12–18 hours before the surgery to reduce the risk of aspiration during surgery. Ewes were given an intramuscular injection of the antibiotic oxytetracycline (20 mg/Kg Phoenix Pharm, Auckland, New Zealand) 30 minutes before surgery for prophylaxis. Anaesthesia was induced by an i.v. injection of propofol (5 mg/kg, AstraZeneca, Auckland, New Zealand), and maintained with 2 to 3% isoflurane in oxygen after intubation. The depth of anaesthesia, maternal respiration and heart rate was constantly monitored during surgery by trained anaesthetic staff. Maternal fluid balance was maintained by a continuous i.v. infusion of Plasma-Lyte 148 (∼250 ml/h) (Baxter, Auckland, New Zealand).

All surgical procedures were performed using aseptic techniques, as previously described.^18^ A maternal midline incision was made to expose the uterus, and the fetus was partially exteriorised for instrumentation. A femoral and brachial artery, and one brachial vein were catheterised with saline-filled polyvinyl catheters (SteriHealth, Dandenong South, VIC, Australia) for blood pressure measurement, pre-ductal fetal blood sampling and drug infusion, respectively. An additional catheter was secured to the fetus to measure amniotic fluid pressure. An ultrasound flow probe (3S type, Transonic Systems Inc., New York, USA) was placed around a carotid artery to measure carotid blood flow (CaBF; as an index of global cerebral flow).^19^

A pair of electrodes (AS633-3SSF wire, Cooner Wire, Chatsworth, CA, USA) were placed subcutaneously across the fetal chest to measure fetal electrocardiogram (ECG). Two pairs of electrodes (AS633-7SSF, Cooner Wire) were placed through burr holes on the dura over the parietal parasagittal cortex bilaterally, 5 and 10 mm anterior, 5 mm lateral to the bregma to measure fetal electroencephalographic (EEG) activity. The burr holes were sealed with surgical wax and electrodes fixed in place using cyanoacrylate glue. A reference electrode was placed over the occiput. In a subset of 18 fetuses, small fibre optic probes used for near infrared spectroscopy (NIRS) were placed bilaterally on the skull over the parietal lobe 1.5 cm anterior and 1.5 cm lateral to the bregma and optode prisms were secured with rapid setting dental cement (Rocket Red; Dental Ventures of America Inc., Anaheim, CA, USA). An inflatable silicone occluder (OC16HD, 16 mm, In Vivo Metric, Healdsburg, CA, USA) was loosely placed around the umbilical cord to allow post-surgical occlusion to induce fetal HI.

At the end of surgery, fetal catheters were heparinised (20 U/ml heparin in saline), the fetus was returned to the uterus, and the amniotic fluid lost during surgery was replaced with sterile 0.9% saline (∼500 ml at 39°C). The uterus was closed, and the antibiotic Gentamicin (80 mg, Pfizer, Auckland, New Zealand) was administered into the amniotic sac. All the fetal leads were exteriorised through the maternal flank. The maternal midline skin incision was repaired and infiltrated with a local analgesic: 10 ml 0.5% bupivacaine plus adrenaline (AstraZeneca Ltd., Auckland, New Zealand). A maternal long saphenous vein was catheterised for post-operative care.

After surgery, animals were housed together in individual metabolic cages with access to concentrated pelleted food and water *ad libitum*. Rooms were temperature-controlled (16 ± 1°C, humidity 50 ± 10%) with a 12:12 hour light: dark cycle, with lights on at 06.00 hours. A period of 4–5 days of recovery was allowed before the commencement of experiments. Antibiotics were given i.v. to the ewe daily for 4 days; 600 mg benzylpenicillin sodium (Novartis, Auckland, New Zealand) and 80 mg gentamicin (Pfizer, Auckland, New Zealand). Fetal and maternal vascular catheters were maintained patent by continuous infusion of heparinised saline (20 U/ml at a rate of 0.2 ml/hour). The fetal condition was assessed via continuous computer recordings of all fetal physiological variables (LabVIEW for Windows, National Instruments, Austin, TX, USA), and daily fetal arterial samples taken to monitor pH and blood gases (ABL800 Flex analyser, Radiometer, Auckland, New Zealand), glucose and lactate (YSI 2300 Analyser, YSI Ltd., Yellow Springs, Ohio, USA).

### Physiological recordings

Fetal mean arterial blood pressure (MAP, Novatrans II, MX860; Medex, Hilliard, OH, USA), corrected for maternal movement by subtraction of amniotic fluid pressure, fetal heart rate (FHR) derived from the ECG, CaBF, and EEG were recorded continuously from 24 hours before until 72 hours after HI. Data was stored for offline analysis using custom data acquisition software (LabVIEW for Windows). All pressure signals were low-pass filtered with a fifth-order Butterworth filter with a cut-off frequency at 20 Hz and then digitised at a sampling rate of 512 Hz. The raw ECG signal was filtered with an analogue first-order high-pass filter with a cut-off frequency set at 0.05 Hz and a fifth-order low-pass Bessel filter with a cut-off at 100 Hz and digitised at a sampling rate of 1024 Hz. R-R intervals were extracted from this signal to calculate heart rate. The CaBF signal was low-pass filtered with a second-order Butterworth filter at 0.1 Hz, digitised at 512 Hz.

The EEG signals were amplified 10,000× fold and then processed with a first-order high-pass filter at 1.6 Hz and an analogue fifth-order low-pass Butterworth filter with a cut-off frequency set at 500 Hz. The signal was then filtered by a low-pass filter with a digital IIR Type 2 Chebyshev filter with a cut-off frequency of 128 Hz for analysis of raw EEG waveforms for seizures. The real-time intensity spectra and associated parameters were extracted from four-second epochs. Total EEG power (in microvolts squared (µV^2^)) was calculated on the power spectrum between 1 and 20 Hz. For data presentation, the EEG power was log-transformed (EEG power (dB), 10 × log (power)) to give a better approximation of a normal distribution.^20^ Spectral edge frequency was calculated as the frequency below which 90% of the power was present. EEG amplitude and spectral edge were stored as one-minute averages.

Concentration changes in fetal cerebral deoxyhaemoglobin [Hb], oxyhaemoglobin [HbO2] and cytochrome oxidase [CytOx] were measured as 10 seconds averages using a NIRO-500 spectrophotometer (Hamamatsu Photonics KK, Hamamatsu City, Japan). Changes in the cerebral [HbO2], [Hb] and [CytOx] were calculated from the modified Lambert-Beer law using a previously established algorithm which describes optical absorption in a highly scattering medium.^7,8^ The NIRS measures obtained are relative changes from zero, not absolute changes. Standardisation of the inter-optode distance was used to reduce signal variability within and between animals. The key parameters calculated were the total haemoglobin (HbT) and HbD. HbT = HbO2 + Hb and is an index of total cerebral blood volume at a stable haematocrit. HbD is the difference between the concentrations of HbO2 and Hb (HbD = HbO2 − Hb) and is a volume-weighted average of total cerebral intravascular oxygenation.

### Experimental design

Experiments were conducted at 104–105 days of gestation. Fetuses were randomly assigned to the following groups: sham-HI (Sham-HI, n = 10), HI-saline (HI-Sal, n = 10) or HI-phentolamine (HI-Phento, n = 10). Sham occlusion fetuses received no occlusion. All the occlusions were undertaken between 09:00 and 09:30 hours. Fetuses either received a continuous intravenous infusion of saline or phentolamine (10 mg loading bolus, followed by continuous infusion at 10 mg/h) from 15 minutes to 8 hours after the end of umbilical cord occlusion. The dose of phentolamine used in these studies was titrated to restore carotid blood flow to sham-HI levels during post-HI hypoperfusion. Fetal arterial blood was taken at 30 minutes prior to umbilical cord occlusion, 5 and 17 minutes during occlusion, and then 1, 2, 4, 6, 24, 48 and 72 hours after occlusion for pH and blood gas determination and for glucose and lactate measurements. 72 hours after umbilical cord occlusion, ewes and fetuses were killed with an overdose of pentobarbitone sodium, intravenously administered to the ewe (9 g, Pentobarb 300; Chemstock International, Christchurch, New Zealand). This method is consistent with the Animal Welfare Act of New Zealand. Fetuses were delivered by caesarean section, and fetal weight and sex were determined, and fetal organ weights measured. Fetal brains were perfusion fixed via cannulation of both carotid arteries with 500 ml isotonic heparinised saline (20 U/ml) followed by 1 litre of 10% phosphate-buffered formalin (Global Science, Auckland, New Zealand).

### Immunohistochemistry

Brain tissue was post-fixed by immersion in 10% phosphate-buffered formalin for one week, then processed and embedded in paraffin using standard procedures. 10 µm coronal sections were cut at the level of the mid-striatum (23 mm anterior stereotaxic zero) and dorsal hippocampus (17 mm anterior of stereotaxic zero) using a microtome (Leica Jung RM2035, Leica Microsystems Ltd., Albany, New Zealand), and mounted on chrom-alum and gelatine (Sigma-Aldrich) coated slides and oven dried. Immunohistochemical staining was performed as previously described.^21^ The following primary and secondary antibodies were used: 1:200 rabbit anti-neuronal nuclei monoclonal antibody (NeuN, Ab177487, Abcam, Cambridge, England), 1:200 mouse monoclonal anti-2′3′-cyclic nucleotide 3′-phosphodiesterase (CNPase, Ab6319, Abcam), 1:200 rabbit monoclonal anti-oligodendrocyte transcription factor-2 antibody (Olig-2, Ab109186, Abcam), 1:200 rabbit polyclonal anti-ionized calcium binding adaptor molecule 1 (Iba1) (Ab178680, Abcam) anti-mouse biotinylated monoclonal IgG (BA-9200, Vector Laboratories, Burlingame, California, USA, 1:200) or anti-rabbit biotinylated monoclonal IgG (BA – 1000, Vector Laboratories, 1:200).

Sections taken 17 mm anterior from stereotactic zero were used to analyse neuronal density in the following subcortical regions, cornu amnonis (CA) of the dorsal horn of the anterior hippocampus (divided into CA1/2, CA3, CA4, and dentate gyrus). Striatal neuronal density (including caudate nucleus and putamen) and white matter damage in periventricular and intragyral tracts were analysed on a section taken 23 mm anterior to stereotactic zero. For quantification of surviving neurons (NeuN), immature and mature oligodendrocytes (CNPase), total number of oligodendrocytes (Olig2) and microglia and macrophages (Iba1), regions of interest were imaged at 20 times magnification using light microscopy on a Nikon 80i microscope equipped with a DS-Fi1-U3 camera and NIS Elements Br 4.0 imaging software (Nikon Instruments, Melville, New York, USA). In total, three fields in white matter tracts, seven fields in the striatum (four in the caudate nucleus and three in the putamen) and one field in each subdivision of the hippocampus were imaged in each hemisphere. Numbers of positively labelled cells were quantified using ImageJ software (National Institutes of Health, USA). The average of cell count in each region from both the hemispheres was calculated.

### Data analysis

All physiological and histological analysis was performed blinded to the experimental group using codes for experimental protocols. Offline analysis of fetal physiological parameters was performed with customised LabView-based programs. Data were assessed as hourly averages, with the 24-hour period before the experiment used as a reference to evaluate acute physiological changes associated with the experiment. Log transformed EEG power was normalised to baseline for analysis. EEG transients and seizures were identified visually on the raw EEG. EEG transients were defined as fast spikes (<70 ms) and sharp waves (70 to 250 ms) occurring singly.^7,9,22^ Seizure activity was defined as the appearance of sudden, repetitive, evolving stereotypic waveforms, lasting more than 10 seconds with an amplitude greater than 20 µV.^16,18^ Vascular resistance was calculated as MAP/blood flow.^8^

Based on the variance in subcortical neuronal loss and white matter injury after 25 minute umbilical cord occlusion in a previous study,^21^ a sample size of n = 10/group was estimated to have 90% power to detect a 13% reduction in neuronal count in the striatum, and 7% reduction in oligodendrocyte counts in the PVWM. Statistical analysis was performed using SPSS version 25, SPSS Inc., Chicago, Illinois, USA. There was no mortality in the experimental groups. For the continuously recorded physiological parameters and immunohistochemical data, between group comparisons were performed by two-factor mixed-methods analysis of variance (ANOVA), with time or regions of interest as repeated measures and HI and phentolamine as independent variables. Post-hoc tests were performed when a significant overall effect of group or interaction between group and time was found. Between group comparisons were performed by univariate analysis with the Sidak post-hoc test. Statistical significance was accepted as P < 0.05. Data are presented as mean ± standard deviation.

## Results

### Sex distribution, body and organ weights at post-mortem

The sex distribution was comparable between groups (female:male ratio Sham-HI 5:5, HI-Sal 6:4, HI-Phento 4:6) and there was no difference in post-mortem body or spleen weights between the groups. HI was associated with a reduction in brain weight (Sham-HI 29.3 ± 1.4 g, HI-Sal 25.2 ± 0.8 g, HI-Phento 24.6 ± 0.8 g; P = 0.023 Sham-HI *vs.* HI-Sal, P = 0.009, Sham-HI *vs.* HI-Phento), with no difference between the HI groups.

### Cardiovascular and biochemical parameters during HI

During the pre-HI baseline, there was no difference between the groups in any physiological parameters. HI was associated with immediate bradycardia (average nadir FHR: HI-Sal 64.4 ± 7.8 bpm and HI-Phento 67.5 ± 8.4 bpm), progressive hypotension (mean nadir of blood pressure HI-Sal 11.2 ± 0.8 mmHg, HI-Phento 11.6 ± 0.9 mmHg), hypoxia, hypercapnia, and mixed acidosis with no difference between the HI groups (Supplementary Table 1). The time course of recovery of blood gases and acid-base status after HI was comparable between the groups. In the HI-saline group, blood glucose and lactate concentrations were higher than the sham-HI group until 6 hours post-HI but recovered to baseline levels by 2 hours in the HI-phentolamine group (Supplementary Table 1).

### Post-occlusion fetal heart rate and blood pressure

**Figure 1.**
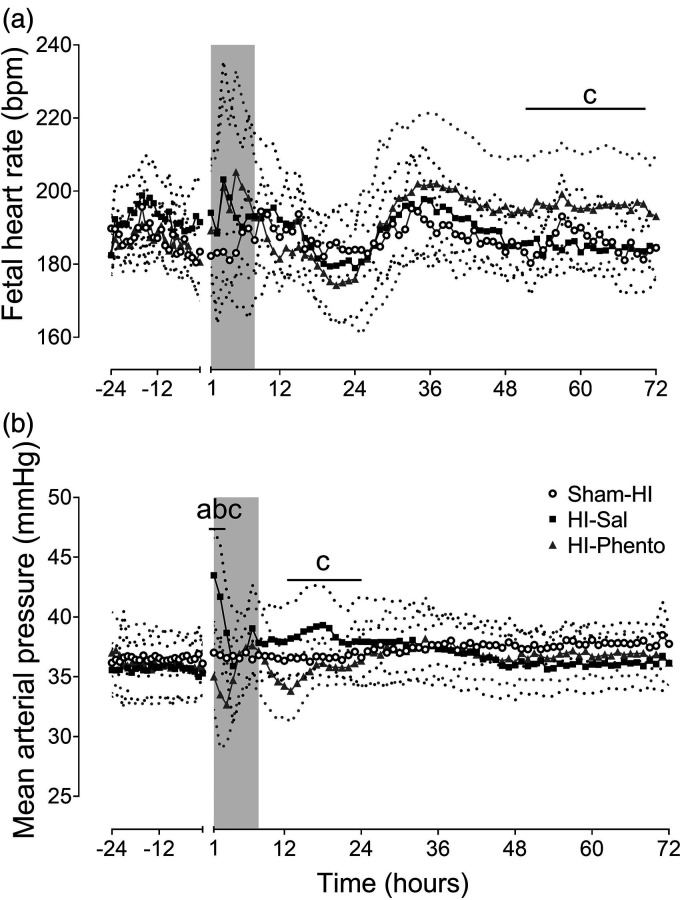
Post-occlusion cardiovascular changes. Time sequence of changes in fetal heart rate (bpm, Panel A) and mean arterial pressure (mmHg, Panel B) during 24 hours before umbilical cord occlusion and 72 hours of post-HI recovery in the sham-HI (open circles, n = 8), HI-saline (closed squares, n = 8) and HI-phentolamine (grey triangles, n = 8) groups. Data during umbilical cord occlusion are not shown here. The shaded area is the period of phentolamine infusion. Data are hourly averages presented as mean ± SD (SD is shown as dotted lines), and were analysed using mixed-design ANOVA with time as a repeated measure and HI and phentolamine as independent factors. Between group comparisons were performed using the Sidak post hoc test. Figure symbols are (a) sham-HI *vs.* HI-saline P < 0.05, (b) sham-HI *vs.* HI-phentolamine P < 0.05 and (c) HI-saline *vs.* HI-phentolamine P < 0.05.

HI was associated with transient tachycardia from 2 to 3 hours post-HI, followed by a reduction in heart rate from 12 to 24 hours, with no significant difference between the groups (Figure 1). FHR in the HI-phentolamine group was higher than the HI-saline group from 48 to 72 hours post-HI (P = 0.046).

MAP was moderately elevated in the HI-saline group from 1 to 3 hours post-HI (P = 0.004 Sham-HI *vs.* HI-Sal) (Figure 1). In contrast, phentolamine was associated with a significant reduction in MAP during early recovery (P = 0.046, Sham-HI *vs.* HI-Phento; P = 0.001, HI-Sal *vs.* HI-Phento, 1 to 3 hours post-HI). MAP in the HI-phentolamine group was significantly lower than the HI-saline group from 12 to 24 hours post-HI (P = 0.019, HI-Sal *vs.* HI-Phento).

### Post-occlusion carotid blood flow and vascular resistance

**Figure 2.**
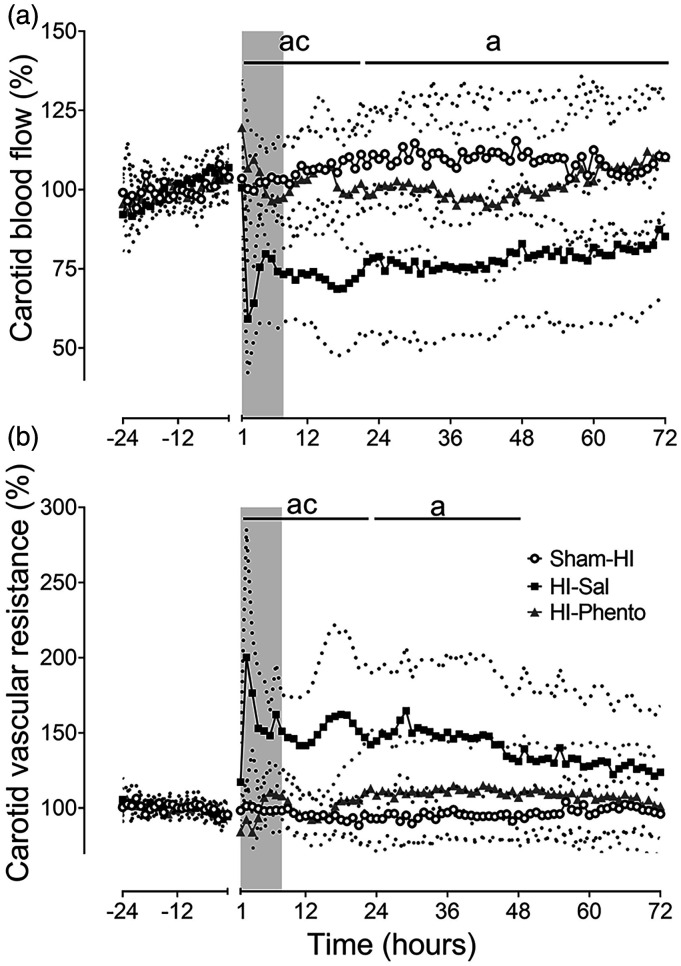
Post-occlusion cerebral blood flow changes. Time sequence of changes in carotid blood flow (% baseline, Panel A) and carotid vascular resistance (% baseline, Panel B) during 24 hours before umbilical cord occlusion and 72 hours of post-HI recovery in the sham-HI (open circles, n = 8), HI-saline (closed squares, n = 8) and HI-phentolamine (grey triangles, n = 8) groups. Data during umbilical cord occlusion are not shown here. The shaded area is the period of phentolamine infusion. Data are hourly averages presented as mean ± SD (SD is shown as dotted lines), and were analysed using mixed-design ANOVA with time as a repeated measure and HI and phentolamine as independent factors. Between group comparisons were performed using the Sidak post hoc test. Figure symbols are (a) sham-HI *vs.* HI-saline P < 0.05, (b) sham-HI *vs.* HI-phentolamine P < 0.05 and (c) HI-saline *vs.* HI-phentolamine P < 0.05.

In the HI-saline group, CaBF transiently recovered to pre-HI baseline values during the first hour after HI, followed by a delayed reduction from 2 hours. CaBF partially recovered from 4 hours but remained significantly lower than the sham-HI group throughout the remainder of the experiment (P = 0.011 Sham-HI *vs.* HI-Sal) (Figure 2). The reduction in perfusion was associated with an increase in carotid vascular resistance (P = 0.001, Sham-HI *vs.* HI-Sal). In the HI-phentolamine group, CaBF was maintained at sham-HI levels throughout the post-HI recovery.

### Neurophysiology

**Figure 3.**
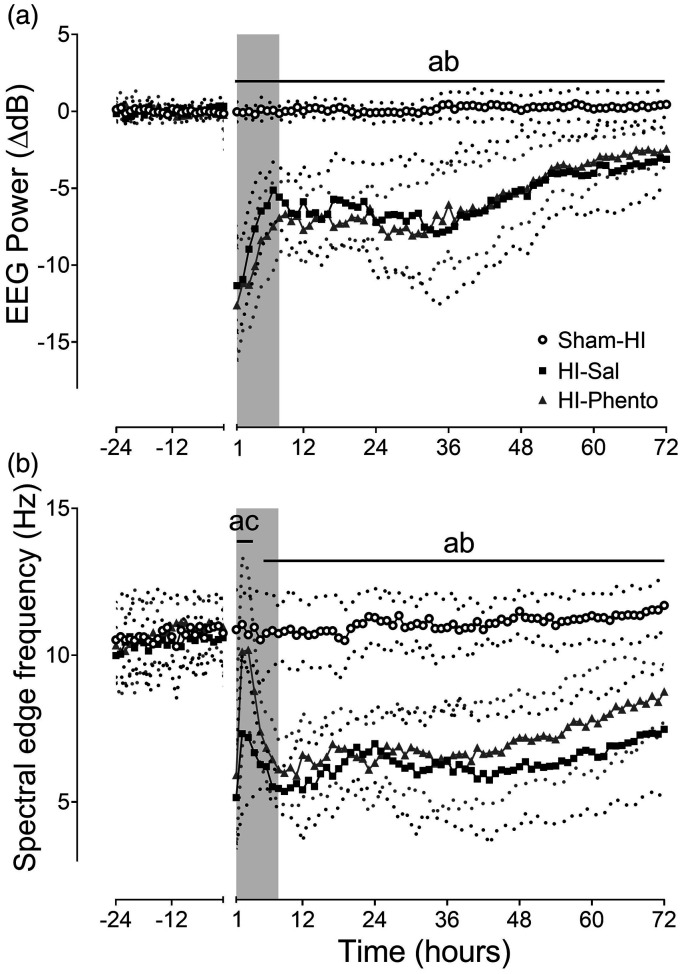
Post-occlusion neurophysiological recovery. Time sequence of changes in EEG power (normalised to baseline ΔdB, Panel A), spectral edge frequency (Hz, Panel B) during 24 hours before umbilical cord occlusion and 72 hours of post-HI recovery in the sham-HI (open circles, n = 10), HI-saline (closed squares, n = 10) and HI-phentolamine (grey triangles, n = 10) groups. Data during umbilical cord occlusion are not shown here. The shaded area is the period of phentolamine infusion. Data are hourly averages presented as mean ± SD (SD is shown as dotted lines), and were analysed using mixed-design ANOVA with time as repeated measure and HI and phentolamine as independent factors. Between group comparisons were performed using the Sidak post hoc test. Figure symbols are (a) sham-HI *vs.* HI-saline P < 0.05, (b) sham-HI *vs.* HI-phentolamine P < 0.05 and (c) HI-saline *vs.* HI-phentolamine P < 0.05.

HI was associated with a rapid suppression of EEG power and spectral edge frequency (Figure 3). EEG power remained suppressed throughout post-HI recovery, with no difference between the occlusion groups (P = 0.001, Sham-HI *vs.* HI-Sal; P = 0.002, Sham-HI *vs.* HI-Phento from 48 to 72 hours post-HI). Between 2 to 4 hours post-HI, there was a transient relative increase in spectral edge frequency that was higher in the HI-phentolamine group than HI-saline group (P = 0.016), and there was no difference between the occlusion groups during the remainder of the recovery (P = 0.001, Sham-HI *vs.* HI-Sal; P = 0.001, Sham-HI *vs.* HI-Phento from 48–72 hours post-HI).

### NIRS measures

**Figure 4.**
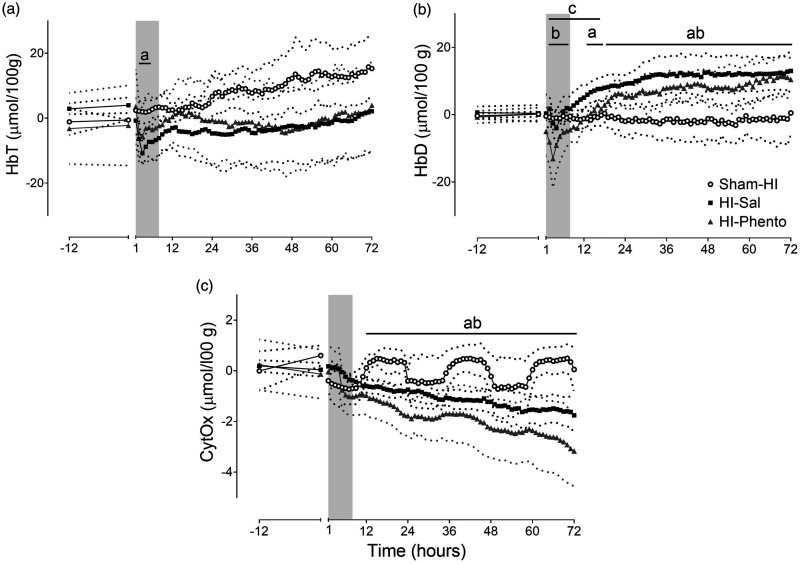
Cerebral oxygenation after HI. Time sequence of changes HbT (µmol/100 g, Panel A), HbD (µmol/100 g, Panel B) and CytOx (µmol/100 g, Panel C) during 12 hours before HI and 72 hours post-HI recovery in the sham-HI (open circles, n = 5), HI-saline (closed squares, n = 8) and HI-phentolamine (grey triangles, n = 5) groups. The shaded area is the period of phentolamine infusion. Data are hourly averages presented as mean ± SD (SD is shown as dotted lines), and were analysed using mixed-design ANOVA with time as a repeated measure and HI and phentolamine as independent factors. Between group comparisons were performed using the Sidak post hoc test. Figure symbols are (a) sham-HI *vs.* HI-saline P < 0.05, (b) sham-HI *vs.* HI-phentolamine P < 0.05 and (c) HI-saline *vs.* HI-phentolamine P < 0.05.

HI was associated with a rapid reduction in HbO2 and increase in Hb that recovered after the end of HI (data not shown). After HI, HbO_2_ (P = 0.042, Sham-HI *vs.* HI-Sal) and HbT fell (P = 0.027, Sham-HI *vs.* HI-Sal) during the first six hours post-HI. There was no effect of occlusion on CytOx concentrations during early recovery. From 12 to 72 hours post-HI, CytOx (P = 0.059, Sham-HI *vs.* HI-Sal) and Hb (P = 0.016, Sham-HI *vs.* HI-Sal) were reduced, and HbD (P = 0.002, Sham-HI *vs.* HI-Sal) was increased in the HI-saline group (Figure 4). Phentolamine infusion altered the early post-HI recovery of NIRS parameters. In the HI-phentolamine group, HbT was comparable to the sham-HI level, but there was a reduction in HbO2 (P = 0.039, Sham-HI *vs.* HI-Phento, not shown) and HbD (P = 0.043, Sham-HI *vs.* HI-Phento, not shown) during the first six hours post-HI. Further, HbD in the HI-phentolamine group was lower than the HI-saline group from 2 to 18 hours post-HI (P = 0.010).

### Seizures

**Figure 5.**
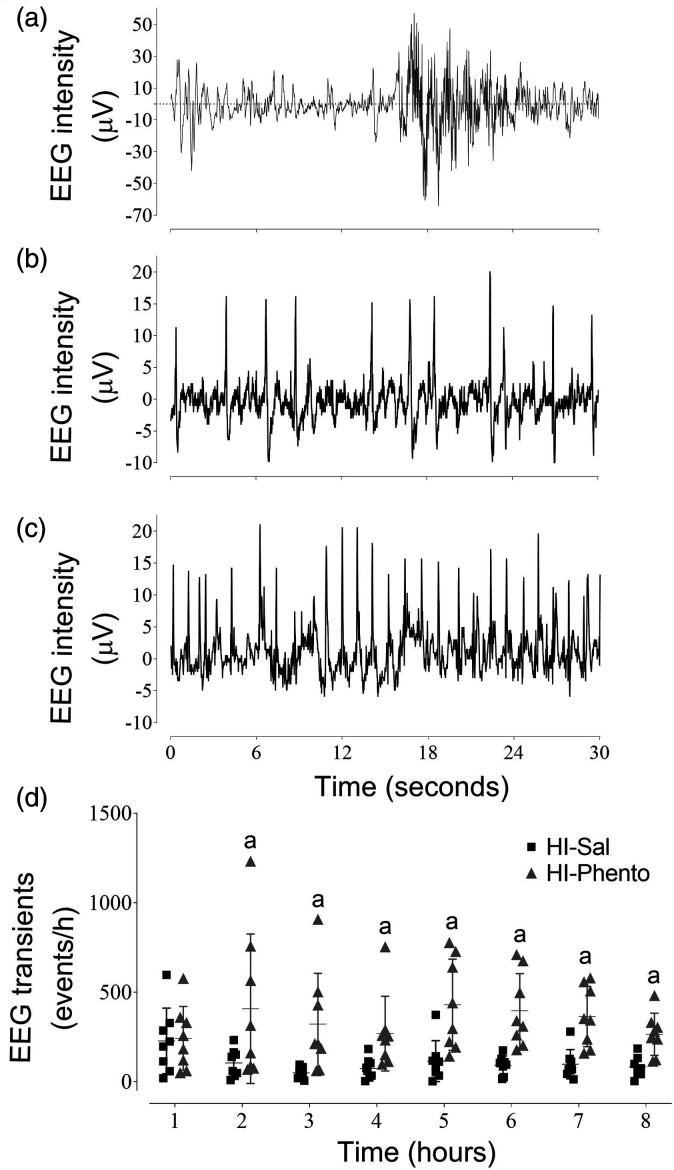
Examples of continuous EEG recordings. EEG recordings from individual fetuses in the sham-HI (Panel A), HI-saline (Panel B) and HI-phentolamine (Panel C) groups at 2 hours after HI. Hourly epileptiform transients counts during the first 8 hours post-HI in the HI-saline (closed squares, n = 8) and HI-phentolamine (grey triangles, n = 8) groups (Panel D). Data are presented as individual animals (the central bar mean ± SD). Figure symbol is (a) HI-saline *vs.* HI-phentolamine P < 0.05.

During early post-HI recovery, a high frequency of epileptiform transients (sharp spikes and waves) was observed on the continuous EEG recording in both the occlusion groups (Figure 5). Phentolamine was associated with an increase in epileptiform transient activity from 2 to 8 hours post-HI (HI-Sal 11.6 ± 5.2 events/minute; HI-Phento 40.9 ± 19.2 event/minute; P = 0.003).

High amplitude stereotypic evolving seizures were not observed in the sham-HI group. These seizures developed on average 7 to 10 hours post-HI in all HI groups. Alpha-adrenergic receptor inhibition with phentolamine did not change the average onset time (HI-Sal 10.9 ± 7.5 hours vs HI-Phento 7.3 ± 2.4 hours) or the total number of seizures (HI-Sal 39.2 ± 23.9 vs, HI-Phento 66.3 ± 43.4), but was associated with a greater number of seizures early after seizure onset. The average seizure count in the HI-phentolamine group was higher than the HI-saline group from 10 to 15 hours post-HI (P = 0.046, supplementary figure 1) . There was no difference between the occlusion groups in the total time spent seizing (HI-Sal 24.2 ± 13.4 hours vs HI-Phento 31.8 ± 17.3 hours), seizure burden (HI-Sal 136.9 ± 69.3 seconds/hour, HI-Phento 144.1 ± 57.9 seconds/hour), average amplitude (HI-Sal 160.5 ± 34.2 µV, HI-Phento 140.7 ± 39.7 µV) and duration (HI-Sal 84.7 ± 38.0 seconds vs HI-Phento 74.3 ± 27.0 seconds) of seizures. In both the HI groups there were two distinct peaks in the average seizure amplitude and duration. The first peak in seizure amplitude and duration was between 12 and 16 hours after the end of HI and the second peak was between 36 and 40 hours. Both the periods of higher average seizure amplitude and duration corresponded with night time (between 9 pm and 1am).

## Immunohistochemistry

### White matter

**Figure 6.**
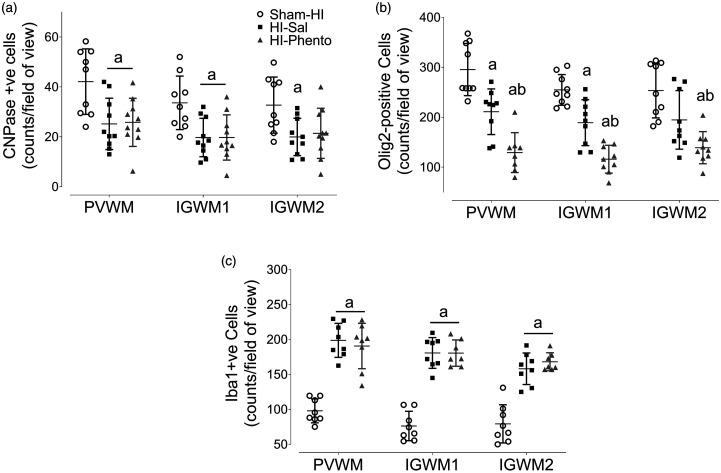
White matter immunohistochemistry. Cell density of immature and mature oligodendrocyte (CNPase, Panel A; Sham-HI n = 9, HI-Sal n = 10, HI-Phento n = 10), total oligodendrocytes (Olig2, Panel B; Sham-HI n = 9, HI-Sal n = 9, HI-Phento n = 9) and microglia and macrophages (Iba1, Panel C; Sham-HI n = 8, HI-Sal n = 8, HI-Phento n = 8) in the white matter areas of periventricular, and parasagittal intragyral areas in the sham-HI (open circles), HI-saline (closed squares), and HI-phentolamine (grey triangles) groups at 72 hours after 25 minutes of umbilical cord occlusion. Data are presented as individual animals (the central bar mean ± SD), and analysed using mixed design two-way ANOVA with regions as repeated measures and HI and phentolamine as independent variables. Comparisons between the groups were performed using the Sidak post hoc test. Figure symbols are (a) P < 0.05 *vs.* sham-HI, (b) P < 0.05 *vs.* HI-saline. PVWM: periventricular white matter, IGWM: intragyral white matter.

HI was associated with a marked reduction in the number of the immature and mature oligodendrocytes (CNPase positive cells) (PVWM P = 0.008 Sham-HI *vs.* HI-Sal, IGWM1 P = 0.009 Sham-HI *vs.* HI-Sal, IGWM2 P = 0.024 Sham-HI *vs.* HI-Sal) and the total number of oligodendrocytes (Olig2 positive cells) (PVWM P = 0.006 Sham-HI *vs.* HI-Sal, IGWM1 P = 0.007 Sham-HI *vs.* HI-Sal, IGWM2 P = 0.001 Sham-HI *vs.* HI-Sal) across all the white matter regions examined (Figure 6 and Supplementary figure 2). Alpha-adrenergic receptor inhibition with phentolamine was associated with a greater loss of the olig2 positive cells (PVWM P = 0.002 HI-Sal *vs.* HI-Phento, IGWM1 P = 0.001 HI-Sal *vs.* HI-Phento, IGWM2 P = 0.034 HI-Sal *vs.* HI-Phento).

HI was associated with a diffuse increase in microglia and macrophages (Iba1-positive cells) in the white matter tracts (PVWM P = 0.001 Sham-HI *vs.* HI-Sal, IGWM1 P = 0.001 Sham-HI *vs.* HI-Sal, IGWM2 P = 0.001 Sham-HI *vs.* HI-Sal) (Figure 6 and Supplementary figure 2). A similar increase in Iba1 positive cells was observed in the HI-phentolamine group, and there was no difference between the occlusion groups (PVWM P = 0.001 Sham-HI *vs.* HI-Phento, IGWM1 P = 0.001 Sham-HI *vs.* HI-Phento, IGWM2 P = 0.001 Sham-HI *vs.* HI-Phento).

### Subcortical neuronal loss

**Figure 7.**
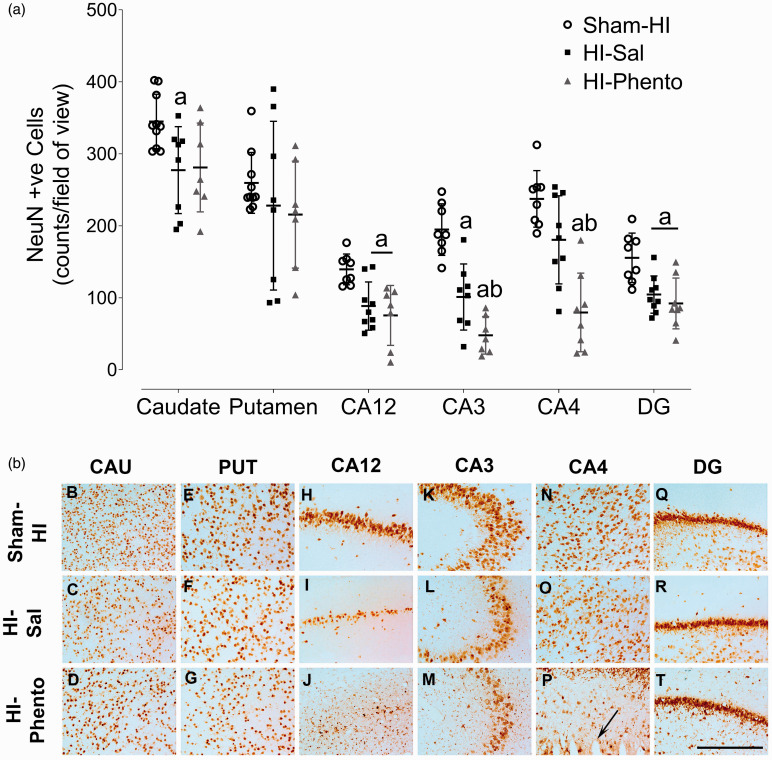
Neuronal survival. Cell density of neurons (labelled with NeuN, Panel A) in caudate nucleus, putamen, CA12, CA3, CA4 and DG of the hippocampus in sham-HI (open circles, n = 10), HI-saline (closed squares, n = 9) and HI-phentolamine (grey triangles, n = 8) groups at 72 hours after 25 minutes of umbilical cord occlusion. Data are presented as individual animals, (central bar is mean ± SD). Data was analysed using two-way ANOVA with region as a repeated measure and HI and phentolamine as independent factors. Comparisons between the groups in each region were performed using the Sidak post hoc test. Figure symbols are (a) P < 0.05 *vs.* sham-HI and (b) P < 0.05 *vs.* HI-saline. CA: cornu amnonis, DG: dentate gyrus. Photomicrographs of neurons (NeuN-positive cells) in the striatal caudate nucleus (caudate, panel B-D), putamen (panel E–G), CA1/2 (panel H–J), CA3 (panel K-M), CA4 (panel N-P) and dentate gyrus (DG, panel Q-T), of the hippocampus of sham-HI, HI-saline and HI-phentolamine groups at 72 hours post-HI. Scale bar is 50 µm.

HI was associated with neuronal loss in the dorsal hippocampus and striatum (CA12 P = 0.021 Sham-HI *vs.* HI-Sal; CA3 P = 0.001 Sham-HI *vs.* HI-Sal; DG P = 0.010 Sham-HI *vs.* HI-Sal; caudate P = 0.032 Sham-HI *vs.* HI-Sal) (Figure 7). Alpha-adrenergic receptor inhibition with phentolamine was associated with increased neuronal loss in the CA3 (P = 0.035 HI-Sal vs. HI-Phento) and CA4 (P = 0.002 HI-Sal vs. HI-Phento) regions of the hippocampus. Further, 50% of the fetuses in the phentolamine infusion group developed infarctions in the CA4 region (Figure 7, Panel P).

## Discussion

The present study demonstrates that an infusion of the non-selective alpha-adrenergic antagonist phentolamine after HI was associated with higher EEG frequency, and increased numbers of epileptiform transients during the latent phase, with a greater fall in cerebral oxygenation during the latent phase, as shown by a greater reduction in HbD, consistent with exacerbation of mismatch between perfusion and metabolism. After 3 days recovery alpha-adrenergic receptor inhibition with phentolamine was associated with greater neuronal loss in the hippocampus and oligodendrocyte loss in the white matter tracts. Together these findings strongly suggest that the SNS plays a key role in actively mediating neuro-inhibition after HI, and that this endogenous neuroinhibition is neuroprotective.

### SNS: post-HI neuro-inhibition and cerebral hypoperfusion

Alpha-adrenergic receptor blockade after HI was associated with increased numbers of epileptiform transients during the latent phase and more seizures during the early secondary phase, with no effect on amplitude and duration, suggesting a reduced threshold for neuronal excitability. Consistent with this, infusion of a specific alpha-2 adrenergic receptor antagonist (idaxozan) after 25-minute umbilical cord occlusion in preterm fetal sheep also increased epileptiform transient activity.^23^ In adult rats exposed to pentylenetetrazole-induced seizures, alpha-2 adrenergic receptor antagonist (atipamezole) increased seizure burden, and alpha-2 adrenergic receptor agonist (dexmedetomidine) reduced hippocampal neuronal activity and seizure burden.^24^ These findings are consistent with alpha-adrenoceptor mediated endogenous neuro-inhibitory modulation.^25^

Phentolamine is a non-specific alpha-adrenergic receptor antagonist. Alpha-adrenoreceptor activation inhibits excitatory synaptic transmission. The alpha-2 adrenoreceptor subtype is the primary mediator of this neuro-inhibitory effect.^25,26^ Ontological studies in sheep from fetus to adult suggested that alpha-1 regulation plays a key role in cerebral vascular development and contractility, and cerebral autoregulation.^27^ Potentially, combined blockade of both alpha-1 and alpha-2 receptor subtypes may have additive effects on neuronal activity.^28^ Alternatively, the alpha-adrenergic receptor subtypes may differentially regulate vascular tone and neuro-inhibition. For example, in the present study phentolamine infusion was associated with both increased neuronal activity and cerebral perfusion, whereas, selective alpha-2 adrenergic receptor blockade after HI in preterm fetal sheep increased epileptiform activity with no effect on secondary hypoperfusion.^23^

Although carotid blood flow was restored to sham-HI level after phentolamine infusion, HbT (an index of cerebral blood volume) was not significantly different between the occlusion groups. Blood haemoglobin concentration was comparable between the occlusion groups, suggesting that HbT values were not affected by differences in haemoglobin levels. The cerebral arterioles of the preterm fetus are innervated by the SNS and sympathetic activation modulates resistance.^29^ Nevertheless, given that the carotid vessels supply extracranial tissues as well as the brain,^30^ we cannot exclude the possibility that improved carotid blood flow during the phentolamine infusion may in part be related to vasodilation of the extracranial beds. It is reasonable to note that previous studies in fetal sheep have shown that carotid blood flow is an index of cephalic blood flow to the brain and correlates well with microsphere and laser Doppler measurements under physiological and pathophysiological conditions,^7,19,30^ and of particularly relevance, laser Doppler and carotid blood flow were highly correlated during delayed post-hypoxic hypoperfusion.^5^ In a previous study, a change of 20% or more in brain blood flow was closely matched by a similar change in carotid arterial flow duration and degree, and changes in oxygenation did not affect the relationship.^30^ It is also important to note that a previous study in 0.6 fetal sheep using radiolabelled tracers reported regional differences in cerebral blood volume within the brain that may not be detected by NIRS recordings from the parietal region.^31^

Despite improving carotid blood flow in the present study, phentolamine was associated with a greater fall in HbD during the latent phase, suggesting exacerbation of the mismatch between cerebral perfusion and metabolism. HbD remained lower than the HI-saline group during the period of increased seizure incidence in the early secondary phase, consistent with ongoing impaired cerebral oxygenation.^32^ HbD is influenced by arterial oxygen saturation, but there was no difference in oxygen saturation and content between the occlusion groups. It is unclear why in the present study perfusion did not increase above sham-HI values to meet the demand associated with increased neural activity. This suggests that there is either a sustained increase in vascular tone mediated by other vasoconstrictors and/or a degree of impaired endothelial function that limited vasodilation. For example, sustained vasoconstriction may, in part be associated with a high expression level of alpha-adrenergic receptors in fetal cerebral arteries and high responsiveness to adrenergic stimulation.^33^

We have previously reported that NMDA receptor activation also contributes to the early phase of delayed hypoperfusion.^34^ In newborn piglets subjected to 10 minutes of ischaemia, infusion of an adenosine uptake inhibitor attenuated delayed cerebral hypoperfusion.^35^ In addition, there is evidence that post-HI endothelial function is impaired by factors such as increased reactive oxygen species (ROS), an altered balance between vasoconstrictors and vasodilators and inflammatory mediators.^12,13^ For example, nitric oxide (NO) is a key vasodilator and the NO synthesis pathway is altered by ROS activity and inflammation, leading to loss of NO bioavailability.^36^

### SNS inhibition: Exacerbation of neural injury

Alpha-adrenergic receptor blockade with phentolamine was associated with greater subcortical neuronal loss and oligodendrocyte loss in the white matter tracts in the present study, strongly supporting an endogenous protective role of alpha-adrenergic receptor activation after HI. Consistent with this, preclinical studies of augmenting the neuroinhibitory effect of the SNS after HI have shown improved neural outcomes. For example, in preterm fetal sheep subjected to 25-minute umbilical cord occlusion, the alpha-2 adrenergic receptor agonist clonidine (10 µg/kg/h, 15 minutes to 4 hours, i.v.) reduced subcortical neuronal loss at 72 hours after HI.^37^ Similarly, in P7 rats, the highly selective alpha-2 adrenergic receptor agonist dexmedetomidine given 1-hour post-HI reduced brain tissue loss at seven days.^38^ However, there are concerns about the dose-dependent adverse effects of alpha-adrenergic agonists.^37,39^

Multiple mechanisms may have contributed to the exacerbation of neural injury in the present study. Firstly, the loss of alpha-adrenergic receptor-mediated endogenous neuro-inhibition leading to transient epileptiform activity may have contributed to greater neuronal loss. Previous studies in preterm fetal sheep have shown that the frequency of epileptiform activity after HI correlates with the severity of hippocampal neuronal loss,^7,23^ and suppression of epileptiform transients was associated with improved subcortical neuronal survival.^34,40^ Increased epileptiform activity may reflect underlying spreading depolarisations, representing a disruption of cellular electrochemical gradient that spreads through various brain regions inducing excessive metabolic demand and intra-cellular calcium overload in the injured tissue.^41,42^ In turn, increased intracellular calcium overload can lead to mitochondrial dysfunction, oxidative stress and activation of the intrinsic cell death pathways.^43^

In addition to the direct effect of phentolamine on neuronal activity, the greater fall in cerebral oxygenation might also contribute to the worsening of neural injury. Post-HI cerebral hypoperfusion is common, and the degree is related to the severity of the insult and normally remains coupled to cerebral metabolic rate with actively mediated vasoconstriction modulating blood flow as in the current study, without hypotension.^5,6,44,45^ We have previously shown that alterations in perfusion-metabolism coupling during post-HI recovery are associated with greater neural injury,^8,46^ for example after exposure to the steroid dexamethasone in preterm fetal sheep.^8,47^ Nevertheless, these findings are correlative, and the absolute magnitude of the fall in HbD was relatively modest (5 µmol/100 g compared to a fall of ∼40 µmol/100 g during profound HI in the same model).^7^ Further studies are needed to quantify the absolute changes in cerebral oxygen consumption.

Phentolamine infusion may have adversely affected mitochondrial function. During post-HI recovery there is a progressive fall in CytOx concentration, as seen in the NIRS data, consistent with a secondary failure of mitochondrial oxidative capacity.^7^ In the HI-phentolamine group, the reduction in CytOx concentration started earlier and was more rapid than in saline treated fetuses, denoting a faster evolution of mitochondrial failure. Consistent with this, there was a significantly greater incidence of seizures after their initial onset, leading to an earlier peak in activity, suggesting a lowered threshold for neuronal excitability after phentolamine exposure. However, it is important to note that high amplitude seizure activity was not altered after the end of phentolamine infusion, including the circadian nature of preterm seizure expression after HI, consistent with our earlier findings.^18^

Interestingly, phentolamine infusion after HI in the present study was associated with increased loss of total oligodendrocytes in the white matter tracts. There was no effect on the survival of immature and mature oligodendrocytes, inferring selective loss of pre-oligodendrocytes, consistent with a developmental vulnerability.^48,49^ Oligodendrocyte progenitor cells express alpha-adrenergic receptors, which play a maturational role.^50,51^ It is not known if the blockade of alpha-adrenergic receptors on oligodendrocytes can directly enhance cell death pathways. Potentially, secondary tissue hypoxia and increased extracellular excitatory amino acids during excessive neuronal activity may have contributed to oligodendrocyte loss after phentolamine infusion. Greater oligodendrocyte loss was not associated with increased microglial activation, suggesting that exacerbation of white matter damage was not mediated by neuroinflammation. Similarly, in adult mice exposed to sepsis, intracerebroventricular injection of an alpha-2 adrenergic antagonist (atipamezole) attenuated the anti-inflammatory and neuroprotective effects of dexmedetomidine but did not increase sepsis-induced microglial activation.^52^

The hippocampus appeared to be particularly vulnerable to greater damage in the phentolamine group. Strikingly, we observed infarction in the CA4 region of the hippocampus in half of the phentolamine group, suggesting that CA4 neurons were particularly susceptible to injury associated with the post-HI increase in epileptiform transients after alpha-adrenergic blockade. Studies in hippocampal slice preparations have demonstrated that hippocampal subfields generate epileptiform activity in a distinct pattern and specific frequency range, and CA4 is associated with the generation of interictal and seizure-like events.^53^ Further, the hippocampal sub-fields show differential responses to pharmacological and endogenous stimuli and to metabolic impairment and hypoxic depolarisation, suggesting different mechanisms are involved in the regulation of regional hippocampal activity.^54,55^

Consistent with the present study, in adult rats the adrenergic neurotoxin (DSP-4) given 2 weeks before cerebral ischemia was associated with exacerbation of post-ischemic neuronal loss in the CA3 and CA4 subfields of hippocampus.^56^ Potentially, a region-specific distribution of alpha-adrenergic receptors could have contributed to the increased vulnerability of these hippocampal regions in the present study. The CA4 region has a high density of adrenergic innervation and of all subtypes of alpha-adrenergic receptors during brain development.^57,58^

## Conclusions

The present study demonstrates that endogenous activation of alpha-adrenergic receptors after HI in preterm fetal sheep inhibits neural activity and reduces neural injury after 3 days recovery. Non-selective alpha-adrenergic receptor blockade increased carotid blood flow, but also increased epileptiform transient activity, with transient worsening of cerebral hypoxia. The specific mechanism of the greater neural injury after alpha-adrenergic receptor blockade is unknown. Potentially, it might reflect the direct loss of endogenous neuroinhibition or an indirect effect through greater exposure to excitotoxicity or reduced tissue oxygenation. Regardless, these data strongly support the concept that alpha-adrenergic receptor activation is a key endogenous neuroprotective mechanism after HI. These data suggest the effects on both cerebral metabolism and blood flow should be considered when investigating potential neuroprotective therapies. Of concern, alpha-adrenergic receptor agonists are increasingly being used as sedatives in critically ill neonates, and can affect oxygenation.^59^ The present study highlights the need for careful assessment of the effects of any intervention on neural activity and cerebral oxygenation.

## Supplemental Material

sj-pdf-1-jcb-10.1177_0271678X231153723 - Supplemental material for Alpha-adrenergic receptor activation after fetal hypoxia-ischaemia suppresses transient epileptiform activity and limits loss of oligodendrocytes and hippocampal neurons Click here for additional data file. Supplemental material, sj-pdf-1-jcb-10.1177_0271678X231153723 for Alpha-adrenergic receptor activation after fetal hypoxia-ischaemia suppresses transient epileptiform activity and limits loss of oligodendrocytes and hippocampal neurons by Simerdeep K Dhillon, Eleanor R Gunn, Mette V Pedersen, Christopher A Lear, Guido Wassink, Joanne O Davidson, Alistair J Gunn and Laura Bennet in Journal of Cerebral Blood Flow & Metabolism

## References

[1] Sarda SP Sarri G Siffel C. Global prevalence of long-term neurodevelopmental impairment following extremely preterm birth: a systematic literature review. J Int Med Res 2021; 49: 030006052110280. 10.1177/03000605211028026 PMC8299900 34284680

[2] Manuck TA Rice MM Bailit JL , et al. Preterm neonatal morbidity and mortality by gestational age: a contemporary cohort. Am J Obstet Gynecol 2016; 215: 103.e1–e14. 10.1016/j.ajog.2016.01.004 PMC4921282 26772790

[3] Dhillon SK Lear CA Galinsky R , et al. The fetus at the tipping point: modifying the outcome of fetal asphyxia. J Physiol 2018; 596: 5571–5592. 29774532 10.1113/JP274949 PMC6265539

[4] Dhillon SK Gunn ER Lear BA , et al. Cerebral oxygenation and metabolism after hypoxia-iFront pharmacolschemia. Front Pediatr 2022; 10: 925951. 35903161 10.3389/fped.2022.925951 PMC9314655

[5] Jensen EC Bennet L Hunter CJ , et al. Post-hypoxic hypoperfusion is associated with suppression of cerebral metabolism and increased tissue oxygenation in near-term fetal sheep. J Physiol 2006; 572: 131–139. 16484307 10.1113/jphysiol.2005.100768 PMC1779646

[6] Michenfelder JD Milde JH Katusic ZS. Postischemic canine cerebral blood flow is coupled to cerebral metabolic rate. J Cereb Blood Flow Metab 1991; 11: 611–616. 2050748 10.1038/jcbfm.1991.111

[7] Bennet L Roelfsema V Pathipati P , et al. Relationship between evolving epileptiform activity and delayed loss of mitochondrial activity after asphyxia measured by near-infrared spectroscopy in preterm fetal sheep. J Physiol 2006; 572: 141–154. 16484298 10.1113/jphysiol.2006.105197 PMC1779651

[8] Lear CA Koome MM Davidson JO , et al. The effects of dexamethasone on post-asphyxial cerebral oxygenation in the preterm fetal sheep. J Physiol 2014; 592: 5493–5505. 25384775 10.1113/jphysiol.2014.281253 PMC4270508

[9] Abbasi H Drury PP Lear CA , et al. EEG sharp waves are a biomarker of striatal neuronal survival after hypoxia-ischemia in preterm fetal sheep. Sci Rep 2018; 8: 16312. 30397231 10.1038/s41598-018-34654-7 PMC6218488

[10] Das Y Leon RL Liu H , et al. Wavelet-based neurovascular coupling can predict brain abnormalities in neonatal encephalopathy. Neuroimage Clin 2021; 32: 102856. 34715603 10.1016/j.nicl.2021.102856 PMC8564674

[11] Schwab AL Mayer B Bassler D , et al. Cerebral oxygenation in preterm infants developing cerebral lesions. Front Pediatr 2022; 10: 809248. 35498781 10.3389/fped.2022.809248 PMC9039301

[12] Amantea D Nappi G Bernardi G , et al. Post-ischemic brain damage: pathophysiology and role of inflammatory mediators. FEBS J 2009; 276: 13–26. 19087196 10.1111/j.1742-4658.2008.06766.x

[13] Spray S Johansson SE Radziwon-Balicka A , et al. Enhanced contractility of intraparenchymal arterioles after global cerebral ischaemia in rat – new insights into the development of delayed cerebral hypoperfusion. Acta Physiol (Oxf) 2017; 220: 417–431. 27864916 10.1111/apha.12834

[14] Patel TR McCulloch J. Failure of an endothelin antagonist to modify hypoperfusion after transient global ischaemia in the rat. J Cereb Blood Flow Metab 1996; 16: 490–499. 8621754 10.1097/00004647-199605000-00016

[15] Bennet L Booth LC Drury PP , et al. Preterm neonatal cardiovascular instability: does understanding the fetus help evaluate the newborn? Clin Exp Pharmacol Physiol 2012; 39: 965–972. 22780577 10.1111/j.1440-1681.2012.05744.x

[16] Quaedackers JS Roelfsema V Heineman E , et al. The role of the sympathetic nervous system in post-asphyxial intestinal hypoperfusion in the preterm sheep fetus. J Physiol 2004; 557: 1033–1044. 15073276 10.1113/jphysiol.2004.062554 PMC1665158

[17] Percie Du Sert N Hurst V Ahluwalia A , et al. The ARRIVE guidelines 2.0: updated guidelines for reporting animal research. J Physiol 2020; 598: 3793–3801. 32666574 10.1113/JP280389 PMC7610696

[18] Bennet L Galinsky R Draghi V , et al. Time and sex dependent effects of magnesium sulphate on post-asphyxial seizures in preterm fetal sheep. J Physiol 2018; 596: 6079–6092. 29572829 10.1113/JP275627 PMC6265534

[19] Gonzalez H Hunter CJ Bennet L , et al. Cerebral oxygenation during postasphyxial seizures in near-term fetal sheep. J Cereb Blood Flow Metab 2005; 25: 911–918. 15729287 10.1038/sj.jcbfm.9600087

[20] Williams CE Gluckman PD. Real-time spectral intensity analysis of the EEG on a common microcomputer. J Neurosci Methods 1990; 32: 9–13. 2335970 10.1016/0165-0270(90)90066-o

[21] Wassink G Davidson JO Dhillon SK , et al. Partial white and grey matter protection with prolonged infusion of recombinant human erythropoietin after asphyxia in preterm fetal sheep. J Cereb Blood Flow Metab 2017; 37: 1080–1094. 27207167 10.1177/0271678X16650455 PMC5363482

[22] Davidson JO Quaedackers JS George SA , et al. Maternal dexamethasone and EEG hyperactivity in preterm fetal sheep. J Physiol 2011; 589: 3823–3835. 21646408 10.1113/jphysiol.2011.212043 PMC3171888

[23] Dean JM Gunn AJ Wassink G , et al. Endogenous alpha(2)-adrenergic receptor-mediated neuroprotection after severe hypoxia in preterm fetal sheep. Neuroscience 2006; 142: 615–628. 16952424 10.1016/j.neuroscience.2006.06.066

[24] Cetindag Ciltas A Ozdemir E Gumus E , et al. The anticonvulsant effects of alpha-2 adrenoceptor agonist dexmedetomidine on pentylenetetrazole-induced seizures in rats. Neurochem Res 2022; 47: 305–314. 34491515 10.1007/s11064-021-03445-4

[25] Hara M Zhou ZY Hemmings HC Jr. alpha2-Adrenergic receptor and isoflurane modulation of presynaptic Ca2+ influx and exocytosis in hippocampal neurons. Anesthesiology 2016; 125: 535–546. 27337223 10.1097/ALN.0000000000001213 PMC4988866

[26] Ohshima M Itami C Kimura F. The alpha2A -adrenoceptor suppresses excitatory synaptic transmission to both excitatory and inhibitory neurons in layer 4 barrel cortex. J Physiol 2017; 595: 6923–6937. 28948610 10.1113/JP275142 PMC5685826

[27] Goyal D Goyal R. Developmental maturation and alpha-1 adrenergic receptors-mediated gene expression changes in ovine middle cerebral arteries. Sci Rep 2018; 8: 1772. 29379105 10.1038/s41598-018-20210-w PMC5789090

[28] Liu W Yuen EY Allen PB , et al. Adrenergic modulation of NMDA receptors in prefrontal cortex is differentially regulated by RGS proteins and spinophilin. Proc Natl Acad Sci U S A 2006; 103: 18338–18343. 17101972 10.1073/pnas.0604560103 PMC1838752

[29] Wagerle LC Kurth CD Roth RA. Sympathetic reactivity of cerebral arteries in developing fetal lamb and adult sheep. Am J Physiol 1990; 258: H1432. 2337177 10.1152/ajpheart.1990.258.5.H1432

[30] Gratton R Carmichael L Homan J , et al. Carotid arterial blood flow in the ovine fetus as a continuous measure of cerebral blood flow. J Soc Gynecol Investig 1996; 3: 60–65. 10.1016/1071-5576(95)00047-X 8796809

[31] Kurth CD Wagerle LC Delivoria-Papadopoulos M. Sympathetic regulation of cerebral blood flow during seizures in newborn lambs. Am J Physiol 1988; 255: H563–H568. 3137827 10.1152/ajpheart.1988.255.3.H563

[32] Soul JS Taylor GA Wypij D , et al. Noninvasive detection of changes in cerebral blood flow by near-infrared spectroscopy in a piglet model of hydrocephalus. Pediatr Res 2000; 48: 445–449. 11004233 10.1203/00006450-200010000-00005

[33] Goyal R Mittal A Chu N , et al. Alpha(1)-adrenergic receptor subtype function in fetal and adult cerebral arteries. Am J Physiol Heart Circ Physiol 2010; 298: H1797–806. 20348219 10.1152/ajpheart.00112.2010 PMC2886655

[34] Dean JM George SA Wassink G , et al. Suppression of post hypoxic-ischemic EEG transients with dizocilpine is associated with partial striatal protection in the preterm fetal sheep. Neuropharmacology 2006; 50: 491–503. 16376952 10.1016/j.neuropharm.2005.10.017

[35] Gidday JM Kim YB Shah AR , et al. Adenosine transport inhibition ameliorates postischemic hypoperfusion in pigs. Brain Res 1996; 734: 261–268. 8896833

[36] Janaszak-Jasiecka A Siekierzycka A Płoska A , et al. Endothelial dysfunction driven by hypoxia – the influence of oxygen deficiency on NO bioavailability. Biomolecules 2021; 11: 982. 34356605 10.3390/biom11070982 PMC8301841

[37] Dean JM George S Naylor AS , et al. Partial neuroprotection with low-dose infusion of the 2-adrenergic receptor agonist clonidine after severe hypoxia in preterm fetal sheep. Neuropharmacology 2008; 55: 166–174. 18572205 10.1016/j.neuropharm.2008.05.009

[38] Ren X Ma H Zuo Z. Dexmedetomidine postconditioning reduces brain injury after brain hypoxia-ischemia in neonatal rats. J Neuroimmune Pharmacol 2016; 11: 238–247. 26932203 10.1007/s11481-016-9658-9

[39] Ezzati M Kawano G Rocha-Ferreira E , et al. Dexmedetomidine combined with therapeutic hypothermia is associated with cardiovascular instability and neurotoxicity in a piglet model of perinatal asphyxia. Dev Neurosci 2017; 39: 156–170. 28391258 10.1159/000458438

[40] Bennet L Dean JM Wassink G , et al. Differential effects of hypothermia on early and late epileptiform events after severe hypoxia in preterm fetal sheep. J Neurophysiol 2007; 97: 572–578. 17093117 10.1152/jn.00957.2006

[41] Shuttleworth CW Andrew RD Akbari Y , et al. Which spreading depolarizations are deleterious to brain tissue? Neurocrit Care 2020; 32: 317–322. 31388871 10.1007/s12028-019-00776-7 PMC7002178

[42] Dreier JP Lemale CL Kola V , et al. Spreading depolarization is not an epiphenomenon but the principal mechanism of the cytotoxic edema in various gray matter structures of the brain during stroke. Neuropharmacology 2018; 134: 189–207. 28941738 10.1016/j.neuropharm.2017.09.027

[43] Thornton C Leaw B Mallard C , et al. Cell Death in the developing brain after Hypoxia-Ischemia. Front Cell Neurosci 2017; 11: 248. 28878624 10.3389/fncel.2017.00248 PMC5572386

[44] Karlsson BR Grogaard B Gerdin B , et al. The severity of postischemic hypoperfusion increases with duration of cerebral ischemia in rats. Acta Anaesthesiol Scand 1994; 38: 248–253. 8023664 10.1111/j.1399-6576.1994.tb03883.x

[45] Keogh MJ Drury PP Bennet L , et al. Limited predictive value of early changes in EEG spectral power for neural injury after asphyxia in preterm fetal sheep. Pediatr Res 2012; 71: 345–353. 22391634 10.1038/pr.2011.80

[46] Dhillon SK Wassink G Lear CA , et al. Adverse neural effects of delayed intermittent treatment with rEPO after asphyxia in preterm fetal sheep. J Physiol 2021; 599: 3593–3609. 34032286 10.1113/JP281269

[47] Koome ME Davidson JO Drury PP , et al. Antenatal dexamethasone after asphyxia increases neural injury in preterm fetal sheep. PLoS ONE 2013; 8: e77480. 24204840 10.1371/journal.pone.0077480 PMC3799621

[48] Back SA. White matter injury in the preterm infant: pathology and mechanisms. Acta Neuropathol 2017; 134: 331–349. 28534077 10.1007/s00401-017-1718-6 PMC5973818

[49] van Tilborg E de Theije CGM van Hal M , et al. Origin and dynamics of oligodendrocytes in the developing brain: implications for perinatal white matter injury. Glia 2018; 66: 221–238. 29134703 10.1002/glia.23256 PMC5765410

[50] Sanders JD Happe HK Murrin LC. A transient expression of functional alpha2-adrenergic receptors in white matter of the developing brain. Synapse 2005; 57: 213–222. 15986363 10.1002/syn.20174

[51] Papay R Gaivin R Jha A , et al. Localization of the mouse alpha1A-adrenergic receptor (AR) in the brain: alpha1AAR is expressed in neurons, GABAergic interneurons, and NG2 oligodendrocyte progenitors. J Comp Neurol 2006; 497: 209–222. 16705673 10.1002/cne.20992

[52] Mei B Li J Zuo Z. Dexmedetomidine attenuates sepsis-associated inflammation and encephalopathy via central α2A adrenoceptor. Brain Behav Immun 2021; 91: 296–314. 33039659 10.1016/j.bbi.2020.10.008 PMC7749843

[53] Reyes-Garcia SZ Scorza CA Araújo NS , et al. Different patterns of epileptiform-like activity are generated in the sclerotic hippocampus from patients with drug-resistant temporal lobe epilepsy. Sci Rep 2018; 8: 7116. 29740014 10.1038/s41598-018-25378-9 PMC5940759

[54] Alkadhi KA. Cellular and molecular differences between area CA1 and the dentate gyrus of the hippocampus. Mol Neurobiol 2019; 56: 6566–6580. 30874972 10.1007/s12035-019-1541-2

[55] Shao LR Janicot R Stafstrom CE. Na(+)-K(+)-ATPase functions in the developing hippocampus: regional differences in CA1 and CA3 neuronal excitability and role in epileptiform network bursting. J Neurophysiol 2021; 125: 1–11. 33206576 10.1152/jn.00453.2020

[56] Nishino K Lin CS Morse JK , et al. DSP4 treatment worsens hippocampal pyramidal cell damage after transient ischemia. Neuroscience 1991; 43: 361–367. 1656318 10.1016/0306-4522(91)90300-d

[57] Happe HK Coulter CL Gerety ME , et al. Alpha-2 adrenergic receptor development in rat CNS: an autoradiographic study. Neuroscience 2004; 123: 167–178. 14667451 10.1016/j.neuroscience.2003.09.004

[58] Winzer-Serhan UH Raymon HK Broide RS , et al. Expression of alpha 2 adrenoceptors during rat brain development–I. Alpha 2A messenger RNA expression. Neuroscience 1997; 76: 241–260. 8971775 10.1016/s0306-4522(96)00368-5

[59] McPherson C Liviskie CJ Zeller B , et al. The impact of dexmedetomidine initiation on cardiovascular status and oxygenation in critically ill neonates. Pediatr Cardiol 2022; 43: 1319–1326. 35212773 10.1007/s00246-022-02854-8 PMC9296564

