# Supplementary material for: Alpha-adrenergic receptor activation after fetal hypoxia-ischaemia suppresses transient epileptiform activity and limits loss of oligodendrocytes and hippocampal neurons
Source: J Cereb Blood Flow Metab. 2023 Jan 26;43(6):947–61. doi: 10.1177/0271678X231153723 (PMC10196751; doi:10.1177/0271678X231153723)
Supplement: sj-pdf-1-jcb-10.1177_0271678X231153723 - Supplemental material for Alpha-adrenergic receptor activation after fetal hypoxia-ischaemia suppresses transient epileptiform activity and limits loss of oligodendrocytes and hippocampal neurons [file sj-pdf-1-jcb-10.1177_0271678X231153723.pdf]

The sympathetic nervous system contributes to post-hypoxia-ischaemia cerebral hypoperfusion and suppression of neural activity

Simerdeep K. Dhillon[1], Eleanor R. Gunn[1], Mette Pedersen[2], Christopher A. Lear[1], Guido Wassink[1], Joanne O. Davidson[1], Alistair J. Gunn[1], Laura Bennet[1].

[1] Department of Physiology, The University of Auckland, Auckland, New Zealand

[2] Department of Pediatrics, Aarhus University Hospital, Aarhus, Denmark

Supplementary Table 1 Fetal arterial blood pH, partial pressures of oxygen and carbon dioxide, glucose, lactate, oxygen saturation (SO2), oxygen content (ctO2), haematocrit (Hct) and haemoglobin (Hb) values at 1 hour before 25 minute occlusion, 5 and 17 minutes during occlusion, 1, 2, 4, 6, 24, 48 and 72 hours after the end of occlusion. Data are presented as Mean ± SD. Between group comparisons are shown as * P < 0.05 *vs.* sham-HI, # P < 0.05 HI-saline group

	**Baseline**	**5 minutes**	**17 minutes**	**1 hour**	**2 hours**	**4 hours**	**6 hours**	**24 hours**	**48 hours**	**72 hours**
**pH**										
Sham-HI	7.37 ± 0.0	7.37 ± 0.0	7.37 ± 0.0	7.37 ± 0.0	7.37 ± 0.0	7.37 ± 0.0	7.37 ± 0.0	7.37 ± 0.0	7.37 ± 0.0	7.37 ± 0.0
HI-saline	7.38 ± 0.0	7.04 ± 0.0*	6.85 ± 0.0*	7.29 ± 0.0	7.33 ± 0.1	7.39 ± 0.0	7.39 ± 0.0	7.36 ± 0.0	7.36 ± 0.0	7.35 ± 0.0
HI-phentolamine	7.36 ± 0.0	7.05 ± 0.1*	6.87 ± 0.0*	7.32 ± 0.0	7.37 ± 0.0	7.41 ± 0.0	7.39 ± 0.0	7.36 ± 0.0	7.37 ± 0.0	7.37 ± 0.0
**pO2 (mmHg)**										
Sham-HI	25.0 ± 3.3	24.5 ± 3.0	24.2 ± 2.2	24.6 ± 1.8	23.5 ± 2.7	24.1 ± 2.7	24.9 ± 4.0	23.7 ± 3.1	24.3 ± 3.0	23.5 ± 3.9
HI-saline	24.3 ± 3.6	8.4 ± 1.6*	11.1 ± 1.7*	28.5 ± 5.5	25.4 ± 4.1	23.0 ± 5.1	23.3 ± 5.0	25.9 ± 5.7	27.0 ± 5.6	27.0 ± 5.6
HI-phentolamine	24.5 ± 2.9	7.5 ± 2.2*	10.3 ± 3.2*	24.7 ± 4.1	22.8 ± 3.5	24.8 ± 2.7	25.9 ± 3.8	26.8 ± 3.9	28.2 ± 2.8	27.1 ± 3.2
**pCO2 (mmHg)**										
Sham-HI	47.6 ± 1.9	47.5 ± 4.2	45.5 ± 3.2	48.1 ± 2.8	50.0 ± 2.6	49.3 ± 2.9	50.1 ± 2.6	50.3 ± 2.6	49.7 ± 3.9	49.4 ± 3.8
HI-saline	49.3 ± 3.9	100.1 ± 8.4*	133.7 ± 9.4*	51.2 ± 9.4	51.5 ± 8.5	47.6 ± 5.5	49.4 ± 4.5	48.3 ± 3.5	46.3 ± 5.0	49.1 ± 4.8
HI-phentolamine	47.8 ± 1.8	93.6 ± 2.5*	130.7 ± 2.6*	46.4 ± 1.5	46.1 ± 1.9	46.0 ± 1.2	46.0 ± 1.3	45.8 ± 1.1	45.9 ± 0.8	44.5 ± 0.9
**Glucose (mmol/L)**										
Sham-HI	1.0 ± 0.4	1.2 ± 0.3	1.1 ± 0.3	1.1 ± 0.3	1.1 ± 0.3	1.1 ± 0.3	1.2 ± 0.3	1.2 ± 0.2	1.2 ± 0.2	1.1 ± 0.2
HI-saline	1.0 ± 0.2	0.3 ± 0.1*	0.7 ± 0.2*	1.3 ± 0.3	1.4 ± 0.4*	1.4 ± 0.3*	1.4 ± 0.2	1.1 ± 0.2	1.2 ± 0.2	1.1 ± 0.2
HI-phentolamine	0.9 ± 0.1	0.3 ± 0.0*	0.6 ± 0.1*	1.5 ± 0.2	1.0 ± 0.0#	1.0 ± 0.0#	1.0 ± 0.0	1.1 ± 0.1	1.1 ± 0.1	1.1 ± 0.1
**Lactate (mmol/L)**										
Sham-HI	0.7 ± 0.2	0.8 ± 0.1	0.9 ± 0.2	0.9 ± 0.2	0.8 ± 0.2	0.9 ± 0.2	0.9 ± 0.2	0.8 ± 0.1	0.8 ± 0.2	0.8 ± 0.2
HI-saline	0.9 ± 0.3	4.3 ± 0.9*	7.1 ± 0.9*	4.4 ± 1.6*	4.1 ± 2.4*	2.9 ± 1.9*	2.5 ± 1.6*	1.1 ± 0.6	0.9 ± 0.3	0.8 ± 0.2
HI-phentolamine	0.6 ± 0.0*	3.7 ± 0.2*	6.0 ± 0.2*	3.1 ± 0.3*	1.5 ± 0.2	1.0 ± 0.2	1.1 ± 0.2	0.7 ± 0.1	0.7 ± 0.1	0.7 ± 0.1

**SO2**										
Sham-HI	71.4 ± 5.3	72.2 ± 4.3	72.1 ± 4.4	71.0 ± 7.1	69.8 ± 7.9	71.3 ± 5.8	71.6 ± 4.9	69.6 ± 5.8	69.2 ± 3.6	68.5 ± 5.2
HI-saline	64.6 ± 9.1	7.9 ± 1.7*	9.2 ± 2.0*	69.3 ± 14.7	64.7 ± 11.5	61.2 ± 15.7	63.0 ± 16.3	67.0 ± 13.0	69.6 ± 11.2	69.7 ± 11.3
HI-phentolamine	71.2 ± 5.9	8.3 ± 1.6*	8.6 ± 2.1*	68.1 ± 11.7	65.0 ± 12.0	73.6 ± 7.8	75.0 ± 9.5	74.8 ± 10.2	79.6 ± 6.3	77.3 ± 8.7
**ctO2**										
Sham-HI	3.8 ± 0.4	3.8 ± 0.6	3.8 ± 0.4	3.6 ± 0.5	3.6 ± 0.5	3.7 ± 0.4	3.9 ± 0.5	3.6 ± 0.4	3.6 ± 0.5	3.6 ± 0.6
HI-saline	3.5 ± 0.3	0.4 ± 0.1*	0.5 ± 0.0*	4.0 ± 0.6	3.7 ± 0.5	3.3 ± 0.8	3.4 ± 0.8	3.6 ± 0.7	3.6 ± 0.6	3.7 ± 0.5
HI-phentolamine	3.5 ± 0.3	0.4 ± 0.1*	0.5 ± 0.1*	3.5 ± 0.7	3.2 ± 0.3	3.7 ± 0.2	3.9 ± 0.3	4.1 ± 0.5	4.2 ± 0.2	4.5 ± 0.7*
**Hct**										
Sham-HI	25.3 ± 1.2	25.5± 3.0	25.1 ± 2.0	24.8 ± 2.1	25.2 ± 1.2	24.7 ± 1.3	25.0 ± 1.3	25.1 ± 1.6	25.1 ± 2.2	25.0 ± 1.8
HI-saline	27.4 ± 4.4	28.6 ± 4.6	27.8 ± 3.8	29.2 ± 4.7	28.5 ± 4.6	26.8 ± 4.4	27.3 ± 6.0	26.7 ± 4.3	25.0 ± 3.8	25.6 ± 3.4
HI-phentolamine	24.4 ± 2.3	28.1 ± 4.3	27.8 ± 3.7	25.2 ± 2.9	24.4 ± 3.7	24.5 ± 2.3	25.6 ± 2.1	26.8 ± 2.8	26.0 ± 2.0	26.5 ± 3.1
**Hb**										
Sham-HI	8.6 ± 0.4	8.5 ± 0.8	8.5 ± 0.6	8.4 ± 0.7	8.5 ± 0.5	8.4 ± 0.4	8.6 ± 0.4	8.7 ± 0.7	8.6 ± 0.8	8.5 ± 0.7
HI-saline	9.3 ± 1.3	9.7 ± 1.7	9.4 ± 1.4	9.9 ± 1.6	9.6 ± 1.6	9.1 ± 1.5	9.2 ± 2.1	9.1 ± 1.6	8.5 ± 1.3	8.7 ± 1.2
HI-phentolamine	8.3 ± 0.8	9.6 ± 1.4	9.4 ± 1.3	8.6 ± 1.0	8.3 ± 1.2	8.4 ± 0.8	8.6 ± 0.8	9.0 ± 0.9	8.8 ± 0.7	9.0 ± 1.0

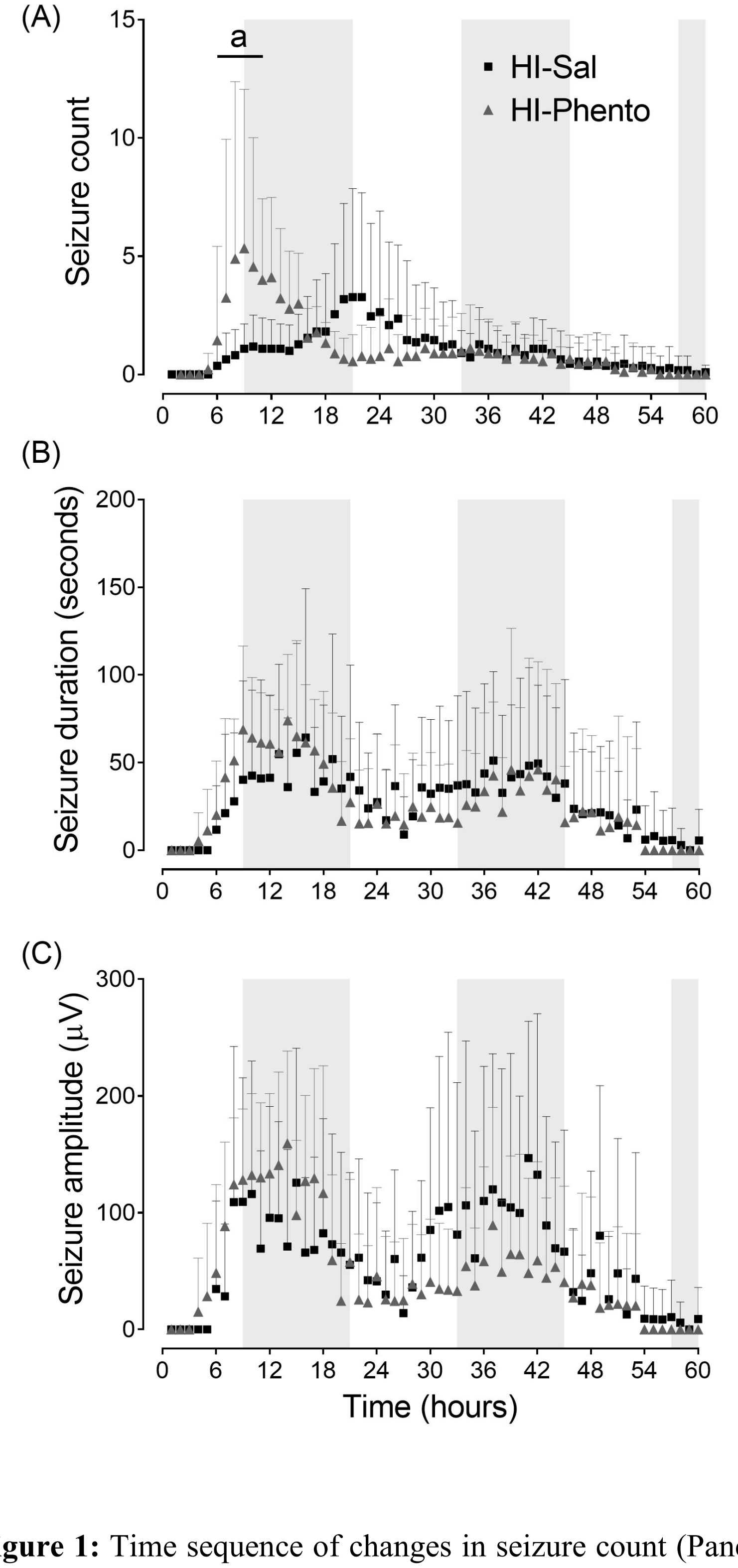

Supplementary Figure 1: Time sequence of changes in seizure count (Panel A), duration (Panel B) and amplitude (Panel C) during the post-HI recovery in the HI-saline (closed squares, n = 9) and HI-phentolamine (grey triangles, n = 10) groups. The shaded areas represent the periods of night time (dark) (6 pm to 6am). Data are hourly averages presented as mean ± SD, and were analysed using one-way ANOVA. Figure symbol is (a) HI-saline vs. HI-phentolamine $P < 0.05$.

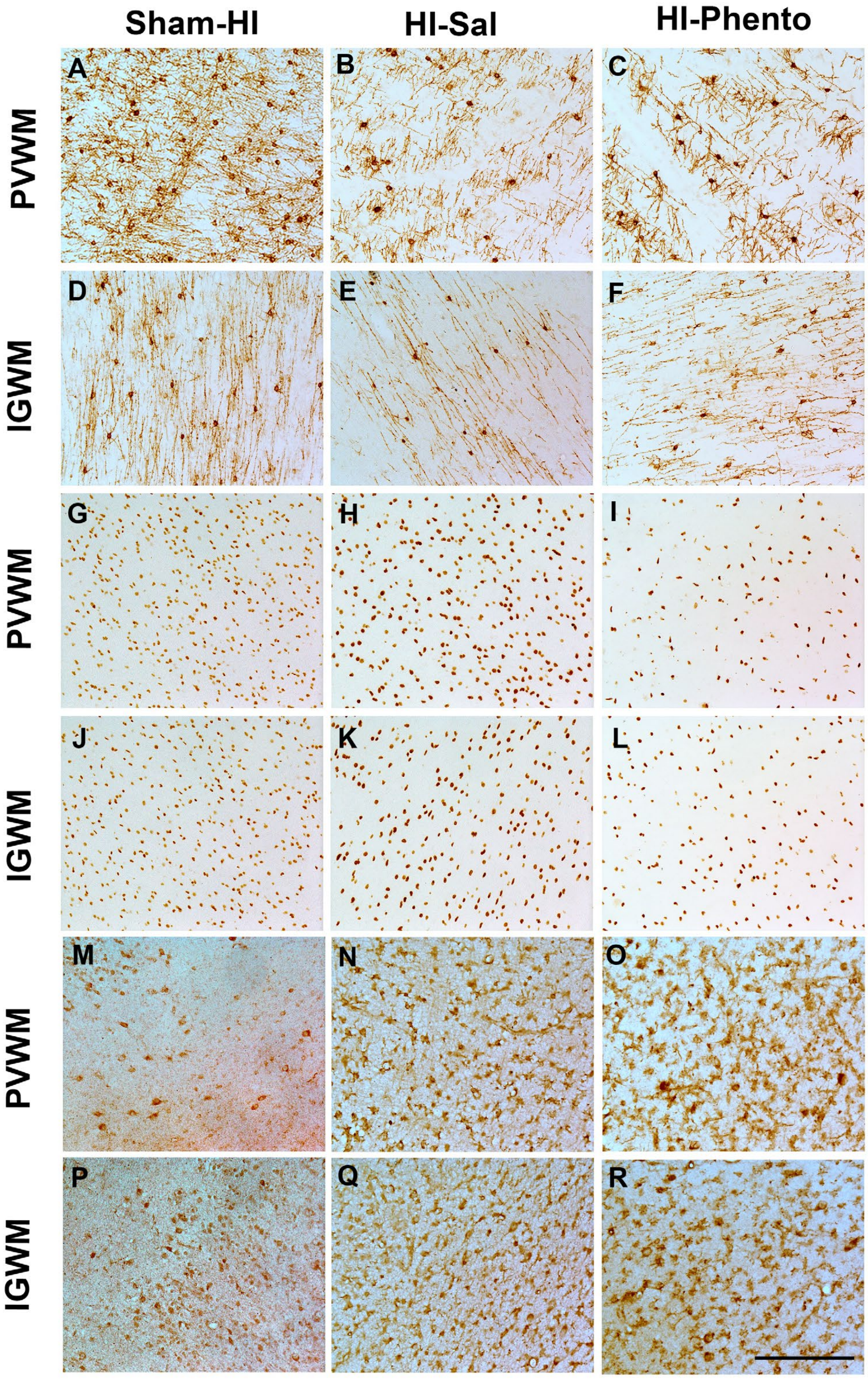

Supplementary Figure 2: Photomicrographs show immature and mature oligodendrocytes (CNPase-positive cells) in PVWM, (Panel A-C), IGWM (Panel D-F), total oligodendrocytes (olig-2-postive cells) in PVWM (Panel G-I) and IGWM (Panel J-L) and microglia and macrophages (Iba1-postive cells) in PVWM (Panel M-O) and IGWM (Panel P-R) in the sham-HI, HI-saline and HI-phentolamine groups at 72 hours post-HI. Scale bar is 50 μm.
